# Harnessing primary, secondary and tertiary genepools for durable wheat disease resistance

**DOI:** 10.1007/s00122-025-05053-0

**Published:** 2025-10-10

**Authors:** Anisa Blower, Rumiana V. Ray, Stephen Rawsthorne, Phil J. Howell, Fiona J. Leigh, Kostya Kanyuka

**Affiliations:** 1 Niab, Park Farm, Villa Rd, Histon, Cambridge, CB24 9NZ UK; 2 University of Nottingham, Plant Sciences Building, Sutton Bonnington Campus, Nottingham, LE12 5RD UK; 3 The Morley Agricultural Foundation, Morley Business Centre, Deopham Road, Morley St Botolph, Wymondham, NR18 9DF UK

## Abstract

Bread wheat (*Triticum aestivum*), a cornerstone of global food security contributing ~ 20% of daily caloric intake, faces increasing vulnerability to rapidly evolving pathogens. This is due in part to a narrowed genetic base following domestication and modern breeding. Wild and ancestral wheat relatives are critical reservoirs of disease resistance genes for breeding new, resilient varieties. This review explores the contributions of primary, secondary, and tertiary genepools of wheat to disease resistance, highlighting loci effective against fungal pathogens that threaten European wheat production. It examines the challenges of alien gene transfer including crossability barriers, hybrid necrosis, and suppressor loci and reviews modern breeding tools such as marker-assisted selection, genomic selection, and genome editing for harnessing exotic germplasm. By synthesising current knowledge, this review highlights the vital contribution of ancestral wheat germplasm in enhancing the resilience and productivity of future wheat crops against increasing biotic stresses.

## *Triticum aestivum*: a globally important cereal

By 2050, the global population is projected to exceed 9.8 billion, leading to a conservative estimate of over 50% increase in overall food demand (Falcon et al. 2022). Food security is defined as “a situation that exists when all people, at all times, have physical, social, and economic access to sufficient, safe and nutritious food that meets their dietary needs and food preferences for an active and healthy life” (FAO 2002). It relies heavily on a small number of staple crops, among which wheat ranks second in global productivity (808.44 million tonnes harvested in 2022) (FAOSTAT 2024).

Wheat (*Triticum* spp.), is crucial in meeting global nutritional demands, providing versatile sources of carbohydrate, fibre and essential nutrients (Shiferaw et al. 2013; Shewry et al. 2013). It contributes approximately 20% of daily caloric intake worldwide. Among wheat species, common or bread wheat (*T. aestivum*), a hexaploid species, is by far the most cultivated, accounting for more than 95% of total global wheat production (de Sousa et al. 2021).

The 'Green Revolution' (1961–1985) underscored the transformative power of crop breeding and agricultural investment in boosting food security. During this period, global cereal productivity tripled despite only a 30% increase in cultivated land area (John and Babu 2021), resulting in improved caloric availability and significant reductions in poverty and food prices. In the UK, wheat yields rose by 126% between 1961 and 2000, driven largely by the adoption of high-yielding semi-dwarf varieties and increased agrochemical inputs (Curtis and Halford 2014).

More recently, however, wheat yields have plateaued in many regions (Cassman and Grassini 2020), raising concerns about the sustainability of current agricultural systems, particularly under the dual pressures of population growth and climate change. Shifts in climatic conditions are expected to influence pathogen dynamics, phenology, and disease pressure, often to the benefit of rapidly evolving pathogens (Burdon and Zhan 2020). This highlights the urgent need for crop improvement strategies that enhance yield resilience while reducing chemical inputs.

### The evolution of modern bread wheat

**Fig. 1:**
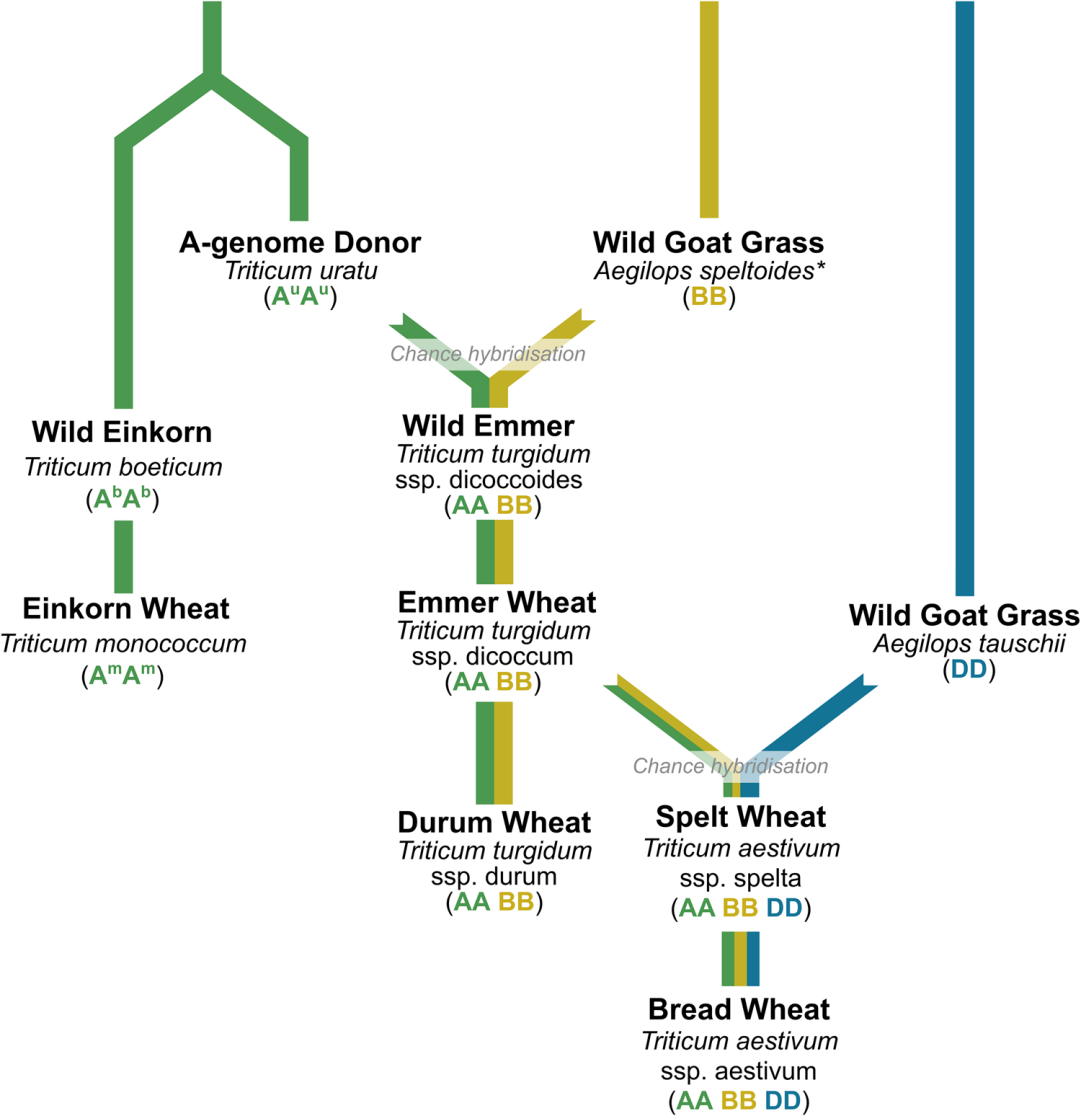
Evolutionary origin of hexaploid bread wheat (*Triticum aestivum*). *Sub-genome* A = green, B = yellow, D = blue, * *extinct diploid species genetically related to Ae. speltoides*

The domestication of wheat traces back more than 10,000 years to the Fertile Crescent in the Middle East (Brown et al. 2009; Weiss and Zohary 2011), where early agricultural societies began cultivating wild grasses, including diploid einkorn (*T. monococcum* ssp. *boeoticum*) and tetraploid emmer (*T. turgidum* ssp. *dicoccoides*). Modern bread wheat, an allohexaploid (AABBDD), is the result of two distinct natural polyploidisation events (Fig. 1).

The first of these events involved hybridisation between a diploid grass *T. urartu* (AA) and a diploid species related to *Aegilops speltoides* (BB) approximately 800,000 years ago (Salse et al. 2008; Levy and Feldman 2022), giving rise to wild emmer wheat (*T. dicoccoides*; AABB)*.* This wild tetraploid lineage remains a valuable source of genetic diversity, including alleles for micronutrient enrichment (Çakmak et al. 2004) and stress resilience (Merchuk-Ovnat et al. 2016; Balla et al. 2022).

One key trait associated with cereal domestication is the loss of a brittle rachis, which enabled seed retention at harvest. In emmer wheat, the genes underlying the *Brittle rachis-A1* (*Br-A1*, *Br2* or *TtBtr1-A*) and *Brittle rachis-B1* (*Br-B1*, *Br3* or *TtBtr1-B*)*,* carry loss-of-function mutations that resulted in non-shattering seed heads and the transition from wild emmer to cultivated emmer (*T. dicoccum*) (Avni et al. 2017). However, its hulled seeds and tough glumes limited early processing efficiency. Continued selection eventually produced durum wheat (*T. durum*), a key tetraploid wheat species with free-threshing seeds and soft glumes and widespread culinary use (Peng et al. 2011).

The second hybridisation event, more recent and rarer, occurred when a cultivated emmer wheat (AABB) hybridised with the diploid goat grass *Ae. tauschii* (DD), producing an undomesticated form of bread wheat (*T. aestivum*; AABBDD) (Fig. 1) (Kihara 1944; Dvořák et al. 1998; Levy and Feldman 2022)*.* Allopolyploidisation, the incorporation of entire genomes from different species, combined traits from multiple progenitor species into a single genome. The resulting enhanced genetic plasticity enhanced tolerance to biotic and abiotic stresses and supported the adaptability and global spread of bread wheat (Dubcovsky and Dvořák 2007).

Modern genotypes have been further refined through human selection for agronomic traits such as reduced plant height, threshability, seed size, and vernalisation requirement (Kilian et al. 2009). However, these selection pressures, combined with historical bottlenecks during domestication and adaptation, have greatly narrowed the genetic base of cultivated wheat (Haudry et al. 2007). This reduction in diversity is believed to underlie recent stagnation in yield gains and increased vulnerability to stress. To reverse this trend, introgression from wild and ancestral relatives is essential for building more resilient and productive wheat varieties.

### Diseases affecting wheat production

Breeding for disease resistance is a cornerstone of sustainable wheat production, especially in the context of climate change, rising pathogen diversity, and the global need to reduce reliance on chemical inputs. Wheat diseases caused by fungal and viral pathogens contribute significantly to yield losses worldwide and pose an ongoing challenge to food security.

**Fig. 2:**
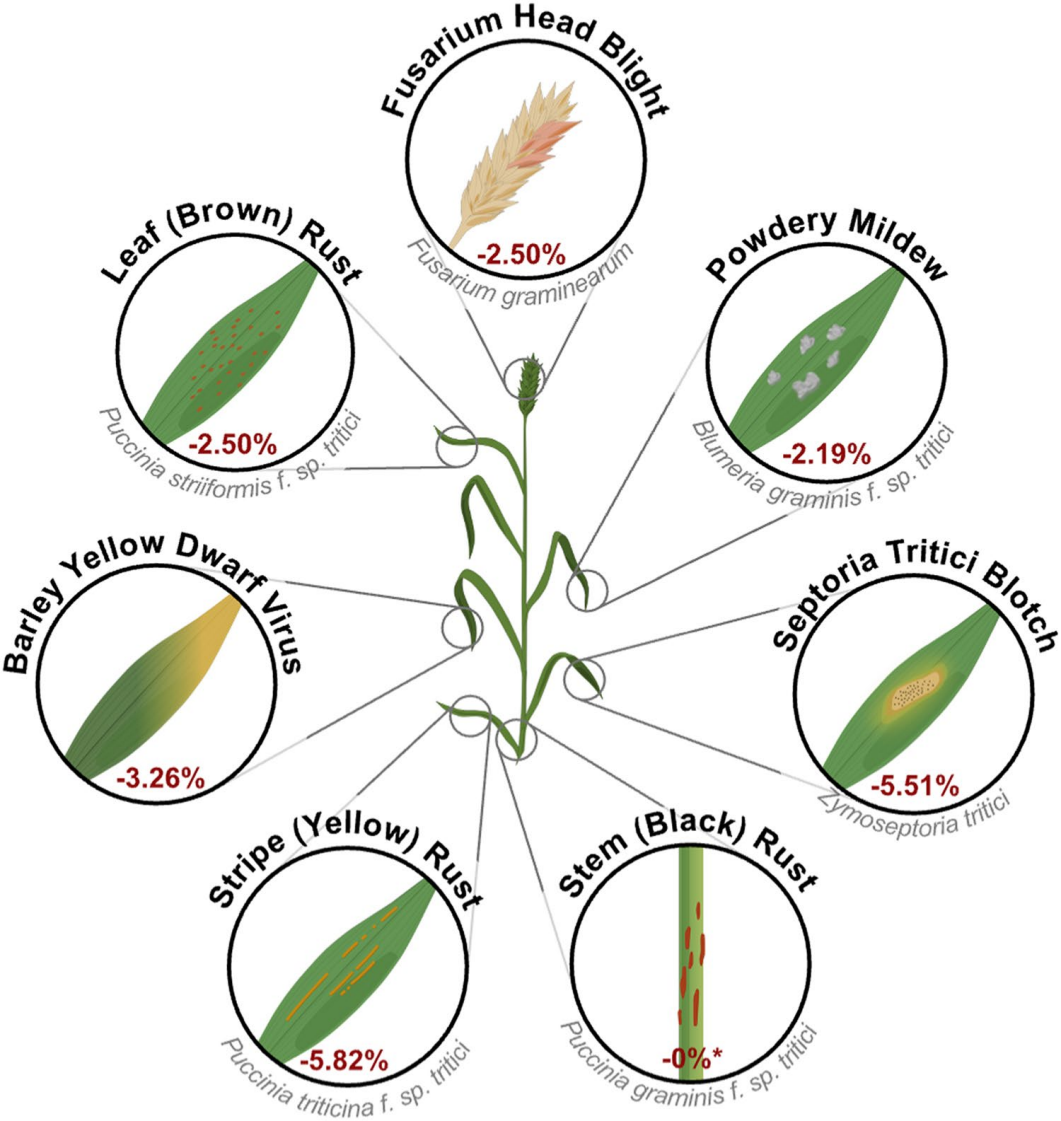
Major diseases affecting wheat production in Northwest Europe. Red values indicate estimated yield losses under current management conditions, including the use of pesticides and deployment of host resistance. Data adapted from Savary et al. (2019) *While stem rust has historically caused minimal losses in this region, its potential resurgence justifies its inclusion among the threats to future wheat production

In Northwest Europe, some of the most damaging diseases include Septoria tritici blotch (STB) (*Zymoseptoria tritici*), yellow rust (*Puccinia striiformis* f. sp. *tritici*), and barley yellow dwarf virus (BYDV). These and others are highlighted in Fig. 2, along with estimated yield losses adapted from global data reported by Savary et al. (2019). While stem rust (*P. graminis* f. sp. *tritici*) has historically been rare in this region, its recent resurgence driven by virulent strains such as the Ug99 lineage and novel European races warrants its inclusion as a disease of future concern. Globally, additional threats include leaf rust (*P. triticina* f. sp. *tritici*), powdery mildew (*Blumeria graminis* f. sp. *tritici*), and Fusarium head blight (FHB) (*Fusarium* species complex), particularly in major wheat-producing regions such as China, India, Russia, USA, Australia, Canada and Europe (Savary et al. 2019; FAOSTAT 2024). FHB reduces grain safety through contamination with mycotoxins such as deoxynivalenol (DON), posing a serious risk to human and animal health (van der Lee et al. 2015).

Although modern varieties carry disease resistance (R) genes, these are frequently overcome by rapidly evolving pathogen populations. The rust fungi, in particular, are known for their capacity to mutate and recombine, giving rise to new virulent strains that can circumvent single R genes within just a few years of their deployment. Of increasing concern is the re-emergence of stem rust in parts of Europe and the Middle East (Lewis et al. 2018; Patpour et al. 2022). Once thought to be largely controlled through resistant varieties and eradication of alternate hosts (e.g. barberry) (Stakman 1923), recent outbreaks in countries such as the UK, Germany, and Italy suggest that stem rust is re-establishing itself in temperate climates. The emergence of aggressive and genetically diverse lineages, including members of the Ug99 race group highlights the urgency of developing new, more durable forms of resistance (Lewis et al. 2024).

This review explores the role of wild and ancestral wheat in enhancing resistance to fungal pathogens of major concern in Europe, with a particular focus on the primary, secondary, and tertiary wheat improvement genepools. We summarise key resistance loci that have been successfully deployed or identified from wild relatives and discuss their potential for future breeding strategies under increasing disease pressure and climate variability. For example, wild and ancestral wheat species, such as *T. dicoccoides*, *T. monococcum*, and *Ae. tauschii*, represent rich and largely untapped reservoirs of R genes. Several valuable resistance loci, including *Yr15* (Randhawa et al. 2009), *Sr22* (Randhawa et al. 2019), and *Stb16q* (Saintenac et al. 2021), have already been introgressed from these sources into modern varieties. Continued exploitation of these wild relatives is essential for expanding the genetic base of resistance and ensuring the resilience of future wheat varieties.

## Primary genepool

The primary genepool of wheat includes species that share fully homologous genomes (A, B, and/or D) with modern bread wheat. This includes hexaploid wheat landraces, tetraploid species such as *T. durum* and *T. dicoccum*, and diploid progenitors such as *T. urartu*, *T. monococcum*, and *Ae. tauschii*. These taxa are generally sexually compatible with bread wheat and can often be crossed without requiring cytogenetic interventions. However, efficient gene transfer, particularly from diploid relatives *T. urartu* and *Ae. tauschii*, often requires the use of synthetic hexaploid wheat (SHW) or high-crossability wheat lines to overcome pre- or post-fertilisation barriers. Despite these limitations, the primary genepool remains the most accessible and widely utilised source of novel allelic variation for crop improvement.

### Landraces and primitive wheats

Hexaploid landraces of bread wheat and wild relatives such as *T. spelta*, *T. macha*, *T. vavilovii*, and *T. sphaerococcum* carry the full AABBDD genomic complement and are fully cross-compatible with modern bread wheat. These underutilised resources have already contributed multiple leaf rust (*Lr*), yellow rust (*Yr*), and powdery mildew (*Pm*) resistance genes. Notably, landraces have been the origin of adult-plant resistance (APR) loci such as *Lr34/Yr18/Pm38* (Krattinger et al. 2009) and *Lr67/Yr46/Pm46* (Moore et al. 2015), which confer durable broad-spectrum, non-race specific resistance to multiple pathogens. Race-specific genes such as *Pm3b/c* have also been traced to *T. aestivum* landraces (Kaur 2008). Efforts to harness this genetic diversity continue through resources such as the Watkins collection, a diverse assembly of global wheat landraces, currently being mined for novel disease resistance alleles and other agronomically valuable traits (Burt et al. 2014; Wingen et al. 2014; Cheng et al. 2024).

*T. spelta* has provided valuable stripe and leaf rust R genes including *Yr5*, *Lr44*, *Lr65*, and *Lr71* (Dyck and Sykes 1994; Singh et al. 2013). Other wild hexaploids remain less explored but hold promise: *T. macha* has contributed quantitative trait loci (QTLs) for FHB resistance on chromosomes 2A, 2B, 5A, and 5B (Buerstmayr et al. 2011); *T. sphaerococcum* carries the stripe rust R gene, *YrSph*, located on 2AS (Chen et al. 2012); and *T. vavilovii* accessions exhibit resistance to rusts and some insect pests (Bariana et al. 2002). A summary of key genes from these sources is provided in Table 1.

**Table 1:** Disease resistance gene contributions from landraces and primitive wheats

Source species	Resistance gene	Donor species chromosome	Reference
*T. aestivum* landrace	*Lr34/Yr18/Pm38*	7D	Krattinger et al. (2009), Lagudah et al. (2009)
*T. aestivum* landrace	*Lr52*	5B	Bansal et al. (2011)
*T. aestivum* landrace	*Lr67/Yr46/Sr55/Pm46*	4DL	Herrera-Foessel et al. (2011), Moore et al. (2015)
*T. aestivum* landrace	*Lr82*	2B	Bariana et al. (2022)
*T. aestivum* landrace	*Pm63*	2BL	Tan et al. (2019)
*T. aestivum* landrace	*Pm59*	7AL	Tan et al. (2018)
*T. aestivum* landrace	*Pm3* alleles	1A	Kaur (2008)
*T. aestivum* landrace	*Yr10*	1BS	Metzger and Silbaugh (1970)
*T. aestivum* landrace	*Yr47*	5BS	Bansal et al. (2011)
*T. aestivum* landrace	*Yr51*	4AS	Randhawa et al. (2014)
*T. aestivum* landrace	*Yr63*	7BS	Mackenzie et al. (2023)
*T. aestivum* landrace	*Yr72*	2BL	Chhetri et al. (2023)
*T. aestivum* landrace	*Yr80*	3BL	Nsabiyera et al. (2018)
*T. aestivum* landrace	*Yr81*	6A	Gessese et al. (2019)
*T. aestivum* landrace	*Yr82*	3BL	Pakeerathan et al. (2019)
*T. aestivum* landrace	*Sr49*	5BL	Bansal et al. (2015)
*T. spelta*	*Lr44*	1B	Dyck and Sykes (1994)
*T. spelta*	*Lr65*	2AS	Mohler et al. (2012)
*T. spelta*	*Lr71*	1B	Singh et al. (2013)
*T. spelta*	*Yr5*	2BL	Macer (1966), Yan et al. (2003)
*T. sphaerococcum*	*YrSph*	2AS	Chen et al. (2012)

### The AB-genome: tetraploid wheats

Tetraploid species such as *T. durum* (durum wheat), *T. dicoccum* (domesticated emmer), *T. dicoccoides* (wild emmer), and *T. carthlicum* (Persian wheat) possess the AABB-genome, enabling full compatibility with hexaploid wheat. These species have played a central role in introducing novel disease resistance alleles into modern breeding programmes (Table 2).

**Table 2:** Disease resistance gene contributions from tetraploid wheats

Source species	Resistance gene	Donor species chromosome	Reference
*T. turgidum* ssp. *carthlicum*	*Pm33*	2BL	Zhu et al. (2005)
*T. turgidum* ssp. *dicoccoides*	*Lr53*	6BS	Marais et al. (2005)
*T. turgidum* ssp. *dicoccoides*	*Lr64*	6AS	Kolmer (2019)
*T. turgidum* ssp. *dicoccoides*	*Pm16*	4A.5BS	Reader et al. (1991), Chen et al. (2005)
*T. turgidum* ssp. *dicoccoides*	*Pm26*	2BS	Rong et al. (2000), Zhu et al. (2025)
*T. turgidum* ssp. *dicoccoides*	*Pm36*	5BL	Blanco et al. (2008)
*T. turgidum* ssp. *dicoccoides*	*Yr15*	1BS	Gerechter-Amitai et al. (1989)
*T. turgidum* ssp. *dicoccoides*	*Yr35*	6BS	Marais et al. (2005)
*T. turgidum* ssp. *dicoccoides*	*Yr84*	1BS	Klymiuk et al. (2022, 2025)
*T. turgidum* ssp. *dicoccoides*	*YrTD121*	1BS	Hu et al. (2025)
*T. turgidum* ssp. *dicoccum*	*Sr2*	3BS	McFadden (1930)
*T. turgidum* ssp. *dicoccum*	*Sr9e*	2B	McIntosh and Luig (1973)
*T. turgidum* ssp. *dicoccum*	*Sr13*	6AL	McIntosh (1972)
*T. turgidum* ssp. *dicoccum*	*Sr14*	1BL	McIntosh (1980)
*T. turgidum* ssp. *dicoccum*	*Sr17*	2BL	McIntosh et al. (1967)
*T. turgidum* ssp. *durum*	*Lr23*	2BS	McIntosh and Dyck (1975)
*T. turgidum* ssp. *durum*	*Lr61*	6BS	Herrera-Foessel et al. (2008)
*T. turgidum* ssp. *durum*	*Lr72*	7BS	Herrera-Foessel et al. (2014)
*T. turgidum* ssp. *durum*	*Lr79*	3BL	Qureshi et al. (2018a)
*T. turgidum* ssp. *durum*	*Sr12*	3BS	Sheen and Snyder (1964)
*T. turgidum* ssp. *durum*	*Sr63*	2AL	Mago et al. (2022)
*T. turgidum* ssp. *durum*	*Srdp2*	6AS	Rondon et al. (1966)
*T. turgidum* ssp. *durum*	*Stb8*	7BL	Adhikari et al. (2003)
*T. turgidum* ssp. *durum*	*Srdp2*	6AS	Rondon et al. (1966)
*T. turgidum* ssp. *durum*	*Stb17*	5AL	Tabib Ghaffary et al. (2012)
*T. turgidum* ssp. *durum*	*Yr7*	2BL	Macer (1966)
*T. turgidum* ssp. *durum*	*Yr30*	3BS	Singh et al. (2000), Wang et al. (2024)
*T. turgidum* ssp. *durum*	*Yr36*	6BS	Uauy et al. (2005)
*T. turgidum* ssp. *durum*	*Yr56*	2AS	McIntosh et al. (2013)
*T. turgidum* ssp. *durum*	*Yr64*	1BS	Cheng et al. (2014)
*T. turgidum* ssp. *durum*	*Yr65*	1BS	Cheng et al. (2014)

Wild emmer wheat (*T. dicoccoides*) has been particularly valuable, contributing both major gene resistance loci and QTLs for complex traits such as FHB and STB (Naz et al. 2015; Soresi et al. 2021). The FHB resistance QTL *Qfhs.ndsu-3AS*, originating from wild emmer, has been fine-mapped for use in durum wheat breeding (Zhu et al. 2016; Soresi et al. 2021). These QTLs offer partial yet durable resistance—particularly important as many major R genes are vulnerable to pathogen evolution.

While *T. dicoccum* remains the most widely utilised tetraploid donor, other species such as *T. carthlicum* and *T. dicoccoides* are gaining attention for their underexploited genetic diversity. The increasing resolution of molecular genetic maps, coupled with advances in genomic selection, are accelerating the identification and deployment of novel R gene loci from these AB-genome sources.

Some tetraploid-derived R genes face suppression in the hexaploid background due to suppressor loci. This phenomenon was first described for stem rust R genes, where *SuSr-D1* on chromosome 7D, encoding the transcriptional regulator MED15 (Hiebert et al. 2020), was shown to suppress certain *Sr* genes from tetraploid donors (Kerber 1964; Kerber and Green 1980)*.* Similar suppression has been observed for the leaf rust resistance genes *LrOft* and *Lr23*, suppressed by *SuLrOft* (2AS) (Zhuansun et al. 2023) and *SuLr23* (2DS) (Nelson et al. 1997), respectively. Understanding and mitigating suppressor effects is essential to ensure effective R gene expression in breeding.

### The A-genome from diploid wheat relatives

Diploid A-genome species such as *T. urartu* (A^u^A^u^) and *T. monococcum* (A^m^A^m^) offer unique resistance loci that are more directly transferable than those from more distantly related species (Table 3). Although their direct use is less common than tetraploids, these species remain valuable for expanding genetic diversity in cultivated wheat.

**Table 3:** Disease resistance gene contributions from diploid A-genome wheat relatives

Source species	Resistance gene	Donor species chromosome	Wheat acceptor chromosome	Reference
*T. boeoticum*	*Pm25*	–	1A	Shi et al. (1998)
*T. boeoticum*	*PmTb7A.1*	–	7AL	Chhuneja et al. (2015)
*T. boeoticum*	*PmTb7A.2* (*Pm1* allele)	–	7AL	Chhuneja et al. (2015)
*T. monococcum*	*LrTm64-8*	2A^m^	2AS	Dinkar et al. (2024)
*T. monococcum*	*Pm1b*	–	7A	Hsam et al. (1998)
*T. monococcum*	*Pm1c*	–	7A	Hsam et al. (1998)
*T. monococcum*	*Pm4d*	–	2AL	Schmolke et al. (2012)
*T. monococcum*	*Pm2026*	5A^m^L	5AL	Xu et al. (2008)
*T. monococcum*	*Sr21*	2A^m^L	2A	The (1973)
*T. monococcum*	*Sr22*	7A^m^	7A	Kerber and Dyck (1973), Olson et al. (2010)
*T. monococcum*	*Sr35*	3A^m^	3A	McIntosh et al. (1984), Zhang et al. (2010)
*T. monococcum*	*Sr60*	5A^m^S	5A	Chen et al.(2018)
*T. monococcum*	*SrTm4*	2A^m^L	2AL	Briggs et al. (2015), Li et al. (2023)
*T. monococcum*	*TmStb1*	7A^m^	7A	Jing et al. (2008)
*T. urartu*	*Pm60,*	7A^u^L	7A	Zou et al. (2018)
*T. urartu*	*PmU*	7A^u^L	7A	Zhang et al. (2018b

*T. urartu*, the A-genome donor of bread wheat, diverged from the direct ancestor of the *T. aestivum* A sub-genome approximately 0.42 million years ago (Wang et al. 2022) but remains highly compatible for gene transfer. Similarly, *T. monococcum*, including its wild (ssp. *boeoticum*) and domesticated (ssp. *monococcum*) forms, harbours resistance to yellow rust, powdery mildew, and STB. Although initial crosses between diploid relatives and elite hexaploid wheat varieties often result in hybrid sterility, fertile progeny can be obtained when using landraces such as Chinese Spring, which carries the recessive allele of *Kr1* gene on chromosome 5B that enhances crossability (Schlegel et al. 2022). Alternatively, *T. monococcum* can be crossed with a bridging species such as *T. durum*, with the resulting F_1_ hybrids subsequently crossed with *T. aestivum* to facilitate gene introgression (Chhuneja et al. 2008; Singh et al. 2010).

Modern approaches such as stable genetic transformation have facilitated the transfer of cloned A-genome R genes such as *Sr35* (Saintenac et al. 2013) and *Sr22* (Steuernagel et al. 2016) into wheat and barley (Bukhari et al. 2020; Hatta et al. 2021). Multi-gene cassettes (e.g. *Sr22-Sr35-Sr45-Sr50-Sr55*) offer the potential for durable resistance without significant fitness penalties, such as reductions in grain yield or plant height (Luo et al. 2021).

Diploid A-genome species are also genetically diverse: a core collection of 19 T*. urartu* and 60 T*. monococcum* accessions captures ~ 98% of known allelic variation (Adhikari et al. 2022). Yet, this diversity remains underexploited in breeding programmes. Advances in genotyping techniques, genomic selection and transgenic technologies, along with genome editing to replicate key wild alleles, provide powerful strategies to harness these valuable genetic resources.

### *Aegilops tauschii* and the D-genome contribution

The D-genome in modern wheat underwent a severe genetic bottleneck during first hybridisation and then domestication, retaining only approximately 14% of the diversity present in its diploid progenitor *Ae. tauschii* (Zhou et al. 2020). As a result, *Ae. tauschii* is a key resource for restoring genetic diversity in wheat, particularly for disease resistance. Large-scale genomic sequencing of *Ae. tauschii* accessions has enabled the discovery of numerous novel resistance genes using innovative methods such as association genetics with R gene enrichment sequencing (AgRenSeq) (Arora et al. 2019). This approach facilitated the rapid cloning of resistance genes *Sr46* and *SrTA1662* (Arora et al. 2019) and re-identified *Sr45* (Steuernagel et al. 2016) and *Sr33* (Periyannan et al. 2013), which had previously been cloned using MutRenSeq and positional cloning, respectively.

While direct crosses between *Ae. tauschii* and bread wheat are often sterile, SHW has enabled the successful incorporation of D-genome alleles. SHWs are produced by crossing tetraploid wheat (AABB) with *Ae. tauschii* to produce sterile ABD triploids (ABD), which are then treated with colchicine to restore fertility and generate stable AABBDD plants (Chèvre et al. 1989). However, hybrid necrosis can limit the success of such crosses—particularly Type II, caused by interactions between *Net1* from the AB-genome of tetraploid wheat and *Net2* from chromosome arm 2DS of *Ae. tauschii,* and Type III, involving *Nec1* from 7DS of *Ae. tauschii* and an unnamed gene from the AB-genome (Nishikawa 1962; Mizuno et al. 2010; Sakaguchi et al. 2016). Though less problematic during SHW creation, Type I hybrid necrosis, caused by an interaction between compatible alleles of *Ne1* from 5BL (Si et al. 2025) and *Ne2* from 2BS (Si et al. 2021) can present barriers in downstream breeding when SHWs are crossed with elite lines (Chu et al. 2006). Given that all three types of hybrid necrosis pose barriers at different stages of SHW development and use, careful parental selection is essential to avoid necrosis-inducing alleles in breeding programmes.

Using genomic data from 242 sequenced *Ae. tauschii* accessions, three distinct lineages were identified, including a rare L3 group originating in Georgia (Gaurav et al. 2022). To capture this diversity, a panel of SHWs have been strategically developed at Niab from genetically diverse and previously unexploited *Ae. tauschii* donors (Ogbonnaya et al. 2013; Gaurav et al. 2022; Wright et al. 2024). These SHWs can be directly crossed with elite bread wheat varieties, provided that appropriate recurrent parents are selected to minimise hybrid necrosis, thereby capturing the diversity of *Ae. tauschii* in an accessible format.

From a nested association mapping population developed using these SHWs and the UK cultivar Robigus, a QTL corresponding to *Yr28*, previously identified in Chinese SHW (Singh et al. 2000), was detected, demonstrating successful introgression of functional yellow rust resistance into UK-adapted backgrounds (Wright et al. 2024). SHWs more broadly have proven highly effective for disease resistance breeding (McFadden and Sears 1946; Das et al. 2016; Morgounov et al. 2018; Lozano-Ramirez et al. 2022) (Table 4). CIMMYT (International Maize and Wheat Improvement Centre) has produced ~ 1000 SHW lines using diverse *Ae. tauschii* accessions (Dreisigacker et al. 2008; Das et al. 2016). Despite this, many *Ae. tauschii* and SHW lines remain underexploited for disease resistance. One success story is the cloning of *Stb16q* from SHW accession Synthetic M3. This cysteine-rich receptor-like kinase (CRK)-encoding gene confers broad-spectrum resistance to STB by inhibiting fungal growth in both the substomatal cavity and apoplast (Saintenac et al. 2021). Unfortunately, while widely deployed in Europe (e.g. in the variety Cellule and its descendants), resistance breakdown has been reported in France, Iran, and Ireland (Dalvand et al. 2018; Kildea et al. 2020; Orellana‐Torrejon et al. 2022).

**Table 4:** Disease resistance gene contributions from *Aegilops tauschii*

Disease	SHW Derived	Non-SHW Derived
Leaf rust	*Lr21*(Rowland and Kerber 1974) *Lr22a* (Hiebert et al. 2007) *Lr32* (Kerber 1987)	*Lr39 (*Raupp et al. 2001*)* *Lr41* (Cox et al. 1994) *Lr42* (Cox et al. 1994) *Lr43* (Cox et al. 1994)
Powdery mildew	*Pm2* (Lutz et al. 1994) *Pm18* (Lutz et al. 1995) *WTK4* (Gaurav et al. 2022)	*Pm34* (Miranda et al. 2006), *Pm35* (Miranda et al. 2007) *Pm58* (Wiersma et al. 2017)
Septoria tritici blotch	*Stb5* (Arraiano et al. 2001) *Stb16q* (Tabib Ghaffary et al. 2012)	
Stem rust	*Sr33* (Casey et al. 2016) *Sr45* (Periyannan et al. 2014)	*Sr46* (Yu et al. 2015) *SrTA1662* (Gaurav et al. 2022)
Yellow rust	*Yr28* (Singh et al. 2000)	

Despite their benefits, SHWs often possess undesirable agronomic traits such as excessive height, delayed flowering, and poor threshability. To retain beneficial traits while eliminating undesirable ones, multiple backcrosses with elite wheat cultivars are required. However, repeated backcrossing can reduce the frequency of beneficial alleles, narrowing genetic diversity and limiting genetic gain. An alternative is synthetic octoploid wheat (AABBDDDD), generated by direct crosses between bread wheat and *Ae. tauschii*, followed by chromosome doubling (Zhang et al. 2018a). This eliminates tetraploid donors, reducing genetic drag while enhancing disease resistance. However, octoploids remain less explored due to concerns over agronomic performance (Zhang et al. 2018a).

## Secondary genepool

**Fig. 3:**
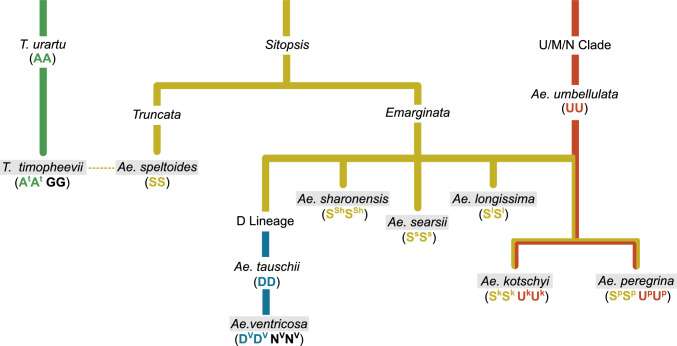
Phylogenetic relationship between species comprising the wheat secondary genepool. Yellow = S-genome lineage, Blue = D-genome lineage, Red = U-genome lineage, Green = A-genome lineage, Dashed arrow = hypothesised geneflow

The secondary gene pool includes species that share at least one homologous genome (A, B, or D) with wheat, enabling partial genetic compatibility (Fig. 3). Key species include polyploids such as *Triticum timopheevii* (A^t^A^t^GG) and *Aegilops ventricosa* (D^V^D^V^N^V^N^V^), along with *Aegilops* species from the Sitopsis section, such as *Ae. speltoides* (SS), which is closely related to the B-genome of wheat. Despite some chromosome pairing between homologous genomes of wheat and these species, structural differences such as translocations and inversions complicate stable gene introgression.

### Facilitating homoeologous pairing

Hexaploid wheat ensures meiotic stability through homoeologous chromosome pairing control (HECP), primarily regulated by the *Pairing homologous gene 1* (*Ph1*) on chromosome 5B. Identified in the 1950s, *Ph1* suppresses non-homologous recombination stabilising meiosis (Okamoto 1957; Riley and Chapman 1958; Martinez et al. 2001; Griffiths et al. 2006). The *ph1b* mutant has been widely used to induce homoeologous recombination, aiding introgression of beneficial traits from wild relatives (Sears 1977; Friebe et al. 2012; Grewal et al. 2018; Li et al. 2020d; Türkösi et al. 2022). Techniques such as the use of the *ph1* mutation, irradiation, and chromosome engineering help overcome these barriers and integrate homoeologous chromosomal segments (Okamoto 1957; Riley and Chapman 1958; Martinez et al. 2001; Griffiths et al. 2006).

Further studies on HECP have revealed additional loci and epigenetic factors influencing recombination, suggesting that gene regulation beyond *Ph1* could provide alternative methods to increase genetic exchange between wheat and wild relatives (Martinez et al. 2001) (Table 5). Recent work has focused on manipulating chromatin structure and histone modifications to enhance pairing potential without disrupting essential genomic stability. Several *Aegilops* species, including *Ae. speltoides* (Feldman and Mello-Sampayo 1967), *Ae. longissima* (Mello-Sampayo 1971), *Ae. peregrina* (syn. *Ae. variabilis*), and *Ae. kotschyi* (Fernández-Calvín and Orellana 1991), naturally harbour *Ph* suppressors that promote homoeologous recombination, offering additional breeding tools. Suppressors such as *Su1-Ph1* (Chr 3S) (Dvořák et al. 2006) and *Su2-Ph1* (Chr 7S) (Dvořák et al. 2006) have been successfully introgressed into wheat, enhancing recombination with wild species and expanding genetic diversity (Li et al. 2017).

**Table 5:** Key genes regulating homoeologous pairing in wheat

Gene	Chromosome	Function
*Ph1*	5B	Suppresses recombination between homoeologous chromosomes
*Ph2*	3D	Minor suppressor of recombination between homoeologous chromosomes
*Su1-Ph1*	3S (*Ae. speltoides*)	Suppresses *Ph1*, thus promoting recombination between homoeologous chromosomes
*Su2-Ph1*	7S (*Ae. speltoides*)	Suppresses *Ph1*, thus promotinges recombination between homoeologous chromosomes

### Gene contributions from *Aegilops* species in the section Sitopsis

*Aegilops* species in the Sitopsis section have significantly contributed to wheat disease resistance. Through chromosome substitution/addition, radiation-induced translocations, and *Ph1* suppression, resistance genes have been introgressed into wheat. *Ae. speltoides* (SS) has provided multiple leaf rust, stem rust and powdery mildew resistance genes (Table 6). While many confer seedling-stage resistance (e.g. *Lr51*, *Sr32*), others offer durable adult-plant resistance (e.g. *Lr28*, *Lr35*, *Lr47*) (McIntosh et al. 1982; Mago et al. 2013). However, introgression efforts are often complicated by gametocidal genes (*Gc1a*, *Gc1b*) on chromosome 2S, which disrupt hybrid fertility (Tsujimoto and Tsunewaki 1988). Research into overcoming gametocidal effects has explored silencing techniques and targeted mutagenesis (Friebe et al. 2003; Zhu et al. 2023). Natural suppressors of gametocidal activity (e.g. *Igc1* in Norin 26) have been identified, which may help in stabilising introgression lines and reducing sterility issues (Tsujimoto and Tsunewaki 1985).

**Table 6:** Resistance gene contributions from *Aegilops* species in the section Sitopsis

Source species	Resistance gene	Donor species chromosome	Wheat acceptor chromosome	Reference
*Ae. longissima*	*Pm13*	3S^l^	3B/3D	Ceoloni et al. (1988)
*Ae. longissima*	*Pm66*	4S^l^	4BL	Li et al. (2020a)
*Ae. longissima*	*PmSl*	6S^l^	6A/6B	Tian et al. (2022)
*Ae. searsii*	*Pm57*	2S^s^	2B	Liu et al. (2017)
*Ae. searsii*	*Sr51*	3S^s^	3DS	Liu et al. (2011a)
*Ae. sharonensis*	*Lr56*	6S^sh^	6A	Marais et al. (2010a)
*Ae. sharonensis*	*Sr62*	1S^sh^	1BL/1DL	Yu et al. (2017)
*Ae. sharonensis*	*Sr-1644-5Sh*	5S^sh^	–	Yu et al. (2017)
*Ae. sharonensis*	*Yr38*	6S^sh^	6A	Marais et al. (2010a)
*Ae. sharonensis*	*Yr87/Lr85*	6S^sh^	6B	Sharma et al. (2024)
*Ae. speltoides*	*Lr28*	7S	4AL	McIntosh et al. (1982)
*Ae. speltoides*	*Lr35*	2S	2B	Kerber and Dyck (1990)
*Ae. speltoides*	*Lr36*	6S	6BS	Dvořák and Knott (1990)
*Ae. speltoides*	*Lr47*	7S	7AS	Dubcovsky et al. (1998)
*Ae. speltoides*	*Lr51*	1S	1B	Dvořák and Knott (1980)
*Ae. speltoides*	*Lr66*	3S	3A	Marais et al. (2010b)
*Ae. speltoides*	*Sr32*	2S	2A	Mago et al. (2013)
*Ae. speltoides*	*Sr39*	2S	2B	Mago et al. (2013)
*Ae. speltoides*	*Sr47*	2S	2B	Faris et al. (2008)
*Ae. speltoides*	*SrAes1t*	2S	2B	Mago et al. (2013)
*Ae. speltoides*	*SrAes7t*	2S	2B	Klindworth et al. (2012)
*Ae. speltoides*	*Pm12*	6S	6B	Miller et al. (1988)
*Ae. speltoides*	*Pm32*	1S	1B	Hsam et al. (2003)
*Ae. speltoides*	*Pm53*	–	5BL	Petersen et al. (2015)

Other *Aegilops* species in the Sitopsis section, including *Ae. sharonensis* (S^Sh^S^Sh^), *Ae searsii* (S^s^S^s^) and *Ae. longissima* (S^l^S^l^) have also contributed valuable resistance genes such as *Lr56*, *Yr38*, *Sr62*, Pm57 and *Pm13* (Table 6). However, linkage drag, the co-introduction of undesirable traits due to tight linkage with a target gene region (Zeven et al. 1983), and recombination suppression have posed challenges. Advances in genetic and cytogenetic techniques, including γ-ray irradiation, have refined translocations to minimise undesirable effects.

### Gene contributions from *Aegilops* species in the section Aegilops

Species like *Ae. kotschyi* (S^k^S^k^U^k^U^k^) and *Ae. peregrina* (S^p^S^p^U^p^U^p^) possess natural repressors of homoeologous recombination, enabling chromosomal exchanges without genetic modifications. Disomic addition lines have facilitated the transfer of resistance genes such as *Lr54* and *Yr37* (*Ae. kotschyi*) and *Lr59* (*Ae. peregrina*). However, large translocations often introduce genetic instability and linkage drag. The *ph1b* mutant (Narang et al. 2018) has been employed to refine these introgressions, facilitating controlled recombination and reducing undesirable traits.

### *Aegilops ventricosa* contributions

Introgressions from *Ae. ventricosa* have provided crucial disease resistance traits (Table 7). Due to ploidy differences, direct crosses with wheat are challenging. *T. turgidum* has historically served as a bridging species, enabling hybrid formation. Two major translocations have been introduced from *Ae. ventricosa*. The first, the 2N^v^S segment, carries the *Yr17–Lr37–Sr38* resistance gene cluster (Bariana and McIntosh 1993), originating from an internal translocation of chromosome arm 6N^v^L (Tanguy et al. 2005). This segment is regarded as one of the most successful alien introgressions after the rye 1BL/1RS translocation, with 29.4% of European wheat cultivars carrying at least one introgressed fragment (Liu et al. 2025). The segment also confers partial resistance to wheat blast (*Magnaporthe oryzae* pathotype *Triticum*), although the effect is background-dependent (Cruz et al. 2016; Cardozo Téllez et al. 2019). The second major contribution is the 7D^v^-derived translocation carrying *Pch1*, a dominant gene for eyespot resistance (Doussinault et al. 1983). *Pch1* has been deployed in cultivars such as Renan, Rendezvous, and Madsen (Hollins et al. 1988; Carter et al. 2020; Langlands-Perry et al. 2021) and remains the most effective source of resistance against the disease (Dumalasová et al. 2015; Pasquariello et al. 2020). Renan and Rendezvous also contain the *Yr17–Lr37–Sr38* gene cluster (Ambrozková et al. 2002). Although *Pch1* introgressions were once linked to yield penalties (Kwiatek et al. 2016), recent work has shown the gene itself is not responsible, and recombinant lines without negative effects have been developed (Pasquariello et al. 2020). These *Ae. ventricosa* segments remain among the most successful alien transfers in wheat breeding.

**Table 7:** Disease resistance gene contributions from *Aegilops ventricosa*

Resistance gene	Donor species chromosome	Wheat acceptor chromosome	Reference
*Lr37*	6N^V^L	2AS	Bariana and McIntosh (1993)
*Pch1*	2N^V^S	2AS	Doussinault et al. (1983)
*Sr38*	6N^V^L	2AS	Bariana and McIntosh (1993)
*Yr17*	6N^V^L	2AS	Bariana and McIntosh (1993)

### *Triticumtimopheevii* contributions

*T. timopheevii* has contributed several disease resistance genes to wheat, predominantly originating from chromosome 2G (Table 8). The A^t^ and A-genomes share homology (Dvořák and Knott 1990), however the G genome, while related to the wheat B-genome (Golovnina et al. 2007), shows restricted recombination. Despite this, genes such as *Pm6*, *Lr50*, and *Sr36* have been successfully introgressed into chromosome 2B (Gordeeva et al. 2009; Timonova et al. 2013; King et al. 2022). The continued exploration of *T. timopheevii* genomic resources has revealed additional resistance loci including for FHB, that may provide untapped genetic potential for wheat improvement (Steed et al. 2022). Efforts to develop near-isogenic lines with defined genomic segments from *T. timopheevii* aim to clarify the effects of introgressions on agronomic traits, disease resistance, and stress tolerance.

**Table 8:** Disease resistance gene contributions from *Triticum timopheevii*

Resistance gene	Donor species chromosome	Wheat acceptor chromosome	Reference
*Lr18*	5G	5B	Dyck and Samborski (1969)
*Lr50*	2G	2B	Brown-Guedira et al. (2003)
*LrSelG12*	3G	3BL	Singh et al. (2017)
*LrTt1*	2A^t^	2A	Leonova et al. (2004)
*LrTt2*	5G	5B	Leonova et al. (2010)
*Pm6*	2G	2B	Joeorgensen and Jensen (1973)
*Pm27*	5G	6B	Järve et al. (2000)
*Pm37*	7A^t^	7A	Perugini et al. (2008)
*Sr36*	2G	2B	McIntosh and Gyarfas (1971)
*Sr37*	4G	4B	McIntosh and Gyarfas (1971)
*Sr40*	2G	2B	Dyck (1992)
*SrTt3*	2G	2B	Gyarfas (1978)

By leveraging advances in genomic sequencing, gene editing, and synthetic hybrid development, the secondary gene pool offers an expanding reservoir of genetic diversity that can significantly benefit wheat breeding in the coming decades.

## Tertiary genepool

The tertiary gene pool includers wild species that are distantly related to wheat. These species lack homologous genomes (A, B, or D) and exhibit limited natural crossability with wheat. Despite these barriers, they represent a valuable source of resistance to biotic and abiotic stresses. Transferring these important traits into wheat requires advanced breeding techniques such as wide hybridisation, the use of bridge species, and chromosome engineering. Notable examples from this genepool include rye (*Secale cereale* L. (RR)) and diploid and polyploid *Thinopyrum* species carrying subgenomes J, E, and St. While integrating genes from these species is challenging, their contributions to wheat improvement have been significant, particularly in enhancing disease resistance and environmental resilience.

### Rye contribution to wheat resistance

Rye stands out as one of the most valuable tertiary gene pool species for wheat improvement. It has provided resistance to several key pathogens (Table 9). However, crossing wheat and rye is not straightforward. Four crossability loci—*Kr1* (5B), *Kr2* (5A), *Kr3* (3D), and *Kr4* (1A) (Zheng et al. 1992)—control hybridisation, with dominant alleles inhibiting crossability by blocking pollen tube growth leading to reduced seed set rates (Lein 1943; Molnár-Láng et al. 2010). Among these, *Kr1* exerts the strongest inhibitory effect. A fifth locus, the suppressor gene, *SKr* (Tixier et al. 1998), located on wheat chromosome 5B, restores crossability in some genotypes (Bouguennec et al. 2018). Recessive *kr* alleles, more common in Chinese and Japanese landraces, facilitate hybridisation, whereas European cultivars predominantly carry dominant *Kr* alleles, limiting crossability. To optimise recombination, geneticists have developed tools like a winter wheat line Mv9kr1*ph1b*. This line, which carries fully recessive *kr* alleles (*kr1kr1kr2kr2)* and the *ph1b* mutation, serves as a “universal bridge” for alien introgressions. For instance, it has enabled the creation of wheat–*Ae. biuncialis* hybrids (Türkösi et al. 2022).

**Table 9:** Disease resistance gene contributions from *Secale cereale*

Resistance gene	Donor species chromosome	Wheat acceptor chromosome	Reference
*Lr25*	2RL	4BL	Procunier et al. (1995), Singh et al. (2012)
*Lr26*	1RS	1BL	Mago et al. (2005b)
*Lr45*	2R	2AS	McIntosh et al. (1995)
*Pm7*	2RL	4B	Heun and Friebe (1990)
*Pm8*	1RS	1BL	Hsam and Zeller (1997)
*Pm17*	1RS	1BL	Hsam and Zeller (1997)
*Pm20*	6R	6BS	Friebe et al. (1994a)
*Pm56*	6RS	6AL	Hao et al. (2018)
*PmCn17*	1RS	1BL	Ren et al. (2009)
*Sr27*	3RS	3AL	Marais and Marais (1994)
*Sr31*	1RS	1BL	Mago et al. (2005b)
*Sr50* (*SrR*)	1RS	1BL	Mago et al. (2004)
*Sr59*	2RL	2DS	Rahmatov et al. (2016)
*Yr9*	1RS	1BL	Mago et al. (2005b)
*Yr83*	6RL	6D	Li et al. (2020b)
*YrCN17*	1RS	1BL	Ren et al. (2009)
*YrR212*	1RS	1BL	Luo et al. (2008)

Despite these challenges, rye has been widely used in wheat breeding through direct introgression and also through the development of novel triticale (× *Triticosecale* Witt.) species with different ploidy levels and genome composition. The most common, hexaploid form (AABBRR), can be used as a bridging species, facilitating the transfer of rye-derived traits into wheat. Chromosomes 1R, 2R, and 3R show full collinearity with their wheat counterparts, whereas 5R and 6R exhibit partial collinearity. In contrast, chromosomes 4R and 7R exhibit highly complex structural rearrangements. Chromosome 4R shows collinearity with sections of multiple wheat chromosomes, while translocations between 7R and wheat 4A have been reported (Li et al. 2021). This significant evolutionary divergence and structural complexity has posed unique challenges for gene transfer in wide hybridisation, limiting the successful transfer of disease resistance genes from these specific chromosomes into cultivated wheat (Table 9).

For over 50 years, rye chromatin introgressions have enhanced wheat resistance to pests and diseases. One of the most famous rye-derived introgressions is the 1BL.1RS translocation, introduced from Petkus rye. This segment carries multiple resistance genes, including *Pm17/Pm8*, *Yr9*, *YrCN17*, *YrR2121*, and *Lr26*. Widely deployed worldwide since the 1960s, this translocation has faced resistance erosion due to pathogen adaptation. For instance, independent virulence mutations in the corresponding pathogen have reduced the effectiveness of *Pm8*, *Yr9,* and *Lr26*, emphasising the need for further genetic diversification (Pretorius et al. 2000; Kunz et al. 2023). Newer 1BL.1RS derivatives from Chinese rye variety Baili exhibit improved resistance to yellow rust pathotypes that overcome *Yr9* (Ren et al. 2022). Beyond 1BL.1RS, additional resistance genes have been identified. For example, *PmTR1* and *PmTR3* (from chromosome 6R) from triticale and rye cultivars Rosen, Prolific, Quinling Insave, Imperial, and Aigan have been utilised in new introgressions. Wild weedy rye species also hold promise. Introgressions from *S. africanum* confer high resistance to yellow rust (Lei et al. 2012, 2013; Li et al. 2016), while *S. vavilovii*, the likely ancestor of cultivated rye (Hagenblad et al. 2016), remains largely unexplored for disease resistance but exhibits high crossability with wheat and may harbour novel resistance genes.

### Wheatgrass species

Wheatgrass species, including *Thinopyrum elongatum* (2*n* = 14, 28, 42, 70), *Th. intermedium* (2*n* = 42), and *Th. ponticum* (2*n* = 70), possess distinct J, E, and St genomes. Their precise genomic relationships remain debated, but their divergence from wheat creates reproductive barriers. Pre-zygotic crossability issues controlled by *Kr1* (5B) and *Kr2* (5A) loci in wheat, prevent fertilisation by alien pollen. Post-zygotic barriers, such as embryo lethality and hybrid sterility, further complicate the process. Additionally, the *Ph1* locus suppresses pairing between homoeologous chromosomes, leading to the transmission of *Thinopyrum* chromatin as large translocations or even whole chromosomes rather than small segments. To overcome these barriers, breeders use bridging crosses, embryo rescue, and treatment with colchicine to prevent hybrid seed abortion in the progeny of wheat–*Thinopyrum* crosses. Despite these challenges, several *Thinopyrum-*derived resistance genes have been successfully transferred into wheat (Table 10).

**Table 10:** Disease resistance gene contributions from *Thinopyrum* species

Source species	Resistance gene	Donor species chromosome	Wheat acceptor chromosome	Reference
*Th. elongatum*	*Cmc2*	6Ag	6DL	Whelan and Hart (1988)
*Th. elongatum*	*Fhb7*	7E	7D	Wang et al. (2020), Wu et al. (2024)
*Th. intermedium*	*Bdv2*	7 J/7S	7D	Stoutjesdijk et al. (2001), Zhang et al. (2004)
*Th. intermedium*	*Bdv3*	7EL	7D	Kong et al. (2009), Ohm et al. (2005)
*Th. intermedium*	*Lr38*	7A^I^	6DL	Friebe et al. (1993), Mebrate et al. (2008)
*Th. intermedium*	*Pm40*	–	7BS	Luo et al. (2009)
*Th. intermedium*	*Pm43*	–	2DL	He et al. (2009)
*Th. intermedium*	*Sr44* (*SrAgi*)	7J^S^	7DS	Liu et al. (2013b), Friebe et al. (1996)
*Th. intermedium*	*Wsm1*	4J^S^S	4DL	Friebe et al. (2009)
*Th. intermedium*	*Wsm3*	7S	7BL	Danilova et al. (2017)
*Th. intermedium*	*Yr50*	–	4BL	Liu et al. (2013a)
*Th. intermedium*	*YrT14*	7 J/7J^S^	6AL/6B	Guo et al. (2023)
*Th. ponticum*	*Lr19*	7E/7Ae	7D	Sarma and Knott (1966), Friebe et al. (1994b)
*Th. ponticum*	*Lr24*	3Ag	3DL	Sears (1973), Schachermayr et al. (1995)
*Th. ponticum*	*Lr29*	7Ae	7DL	Sears (1973), Sibikeev et al. (2022)
*Th. ponticum*	*Pm51*	–	2BL	Zhan et al. (2008), Zhan et al. (2014)
*Th. ponticum*	*Sr24*	3Ae	1BL.1BS/3DL	Smith et al. (1968), Mago et al. (2005a)
*Th. ponticum*	*Sr25*	7E/7Ae	7D	Sarma and Knott (1966), Friebe et al. (1994b)
*Th. ponticum*	*Sr26*	6Ae	6Ae	Qureshi et al. (2018b)
*Th. ponticum/Th. elongatum*	*Sr43*	7EL	7DL	Knott et al. (1977), Yu et al. (2023)
*Th. ponticum*	*YrTp1*	–	2BS	Yin et al. (2006)
*Th. ponticum*	*YrTp2*	–	7BS	Yin et al. (2006)

One of the most notable successes is *Lr19/Sr25*, a resistance gene from *Th. ponticum*, introduced through a wheat-*Thinopyrum* translocation, which remains highly effective against the leaf rust and stem rust pathogens (Sarma and Knott 1966). Similarly, *Sr24* and *Sr26*, also from *Th. ponticum*, provide strong resistance to stem rust, including the highly virulent Ug99 race. *Th. ponticum* itself is a nonhost for certain wheat rust fungi, including stem rust, and wheat lines harbouring its genes exhibit nonhost-type resistance, where fungal infection structures abort early (Plotnikova et al. 2023). Other valuable genes from *Thinopyrum* species include *Bdv2* (Larkin et al. 1995) and *Bdv3* (Ohm and Anderson 2007), which confer resistance to BYDV. Advances in molecular genetics have refined these introgressions, separating desirable genes from linked undesirable ones. For example, the resistance gene *Lr19/Sr25* was successfully uncoupled from the *PSY-E1* gene associated with the undesirable yellow flour pigmentation (Xu et al. 2023).

### Other tertiary genepool contributions

Beyond rye and *Thinopyrum* species, several other distant relatives have contributed resistance genes to wheat (Table 11). Wild species from genera such as *Aegilops*, *Agropyron*, *Dasypyrum*, *Elymus*, and *Leymus* contain valuable genetic variation, though their use is limited by crossability barriers and suppressed recombination. Notable examples include *Lr9*, *Yr40*, and *Sr53* from *Aegilops* species, leaf rust resistance gene *AcRLK2P-1* from *Agropyron cristatum*, *Pm21* and *Sr52* from *Dasypyrum villosum*, *Fhb6* from *Elymus tsukushiensis*, and *Fhb3* from *Leymus racemosus*. Among these, *Pm21* has been most widely used in breeding, particularly in China, where severe powdery mildew pressure has driven its deployment. It has been incorporated into varieties such as Yangmai 5, Yangmai 21, Zhenmai 9, Yangmai 97G59, Yangmai 18, and Yangmai 15, where it confers broad-spectrum resistance (Song et al. 2009; Bie et al. 2015). In contrast, many other alien-derived genes, remain in the research stage due to agronomic trade-offs or incomplete recombination. Advances in genomic tools and marker-assisted selection continue to enhance the accessibility and utility of these alien-derived resistance genes in wheat breeding programs.

**Table 11:** Disease resistance gene contributions from tertiary genepool species

Source species	Resistance gene	Donor species chromosome	Wheat acceptor chromosome	Reference
*A. cristatum*	*AcRLK2P-1*	2PL	6B	Xu et al. (2024)
*A. cristatum*	*Pm-6PL*	6PL	–	Lin et al. (2022)
*Ae. geniculata*	*Lr57*	5M^g^S	5DS	Kuraparthy et al. (2007a)
*Ae. geniculata*	*Pm29*	–	7D	Zeller et al. (2002)
*Ae. geniculata*	*Sr53*	5 Mg	5D	Liu et al. (2011b)
*Ae. geniculata*	*Yr40*	5M^g^S	5DS	Kuraparthy et al. (2007a)
*Ae. neglecta*	*Lr62*	–	6A	Marais et al. (2009)
*Ae. neglecta*	*Yr42*	–	6A	Marais et al. (2009)
*Ae. umbellulata*	*Lr9*	6U	6B	Sears (1956), Schachermayr et al. (1994)
*Ae. umbellulata*	*Lr76* (*LrUmb*)	5U	5D	Bansal et al. (2017, 2020)
*Ae. umbellulata*	*Yr70* (*YrUmb*)	5U	5D	Bansal et al. (2017, 2020)
*Ae. triuncialis*	*Lr58*	2^t^L	2BL	Kuraparthy et al. ( 2007b)
*D. villosum*	*Pm21*	6VS	6AL	Zhu et al. (2018)
*D. villosum*	*Pm55*	5VS	5AL/5DL	Zhang et al. (2016)
*D. villosum*	*Pm62*	2VL	2BS	Zhang et al. (2018c)
*D. villosum*	*Pm67*	1VS	1DL	Zhang et al. (2021a)
*D. villosum*	*Pm4VL*	4 V	7DS	Wei et al. (2024)
*D. villosum*	*Sr52*	6 V	6AS	Qi et al. (2011)
*D. villosum*	*Yr26*	6VS	6AL	Yildirim et al. (2000)
*E. tsukushiensis*	*Fhb6*	1Ets	1AS	Cainong et al. (2015)
*L. racemosus*	*Fhb3*	7Lr	7AS	Qi et al. (2008)

## Technological advances in wheat breeding

Modern plant breeding has moved beyond traditional, subjective methods that relied solely on phenotypic selection. Today, techniques such as marker-assisted selection (MAS), genomic selection (GS), and CRISPR/Cas gene editing (GE) are increasingly integrated into wheat breeding programmes to accelerate the development of disease-resistant cultivars.

### Marker-assisted selection (MAS) and precision breeding

MAS leverages DNA markers tightly linked to genes or QTLs to indirectly select for desirable traits. It remains a cornerstone of modern wheat improvement and is particularly effective for traits controlled by single genes like those conferring resistance to rust, mildew, and FHB. By using tightly linked molecular markers, breeders can introgress these resistance genes without extensive phenotypic screening. MAS is especially powerful for genes with major effects, such as *Lr*, *Yr*, and *Sr* genes, and has been widely used in pyramiding multiple resistance loci into elite wheat varieties.

Marker-assisted backcrossing accelerates the recovery of elite recurrent parent backgrounds by selecting for recombination near the introgressed region and maximum recurrent parent at non-target chromosomes. This approach has been critical in reducing linkage drag associated with alien introgressions, particularly for genes derived from wild relatives such as *Thinopyrum* and *Secale* species.

Successful application of these methods is evident in cases such as wheat variety PBW343 released in 1995 by the Punjab Agricultural University (PAU), India (Sharma et al. 2021). Initially celebrated for its adaptability and yield potential, PBW343 was significantly enhanced through marker-assisted introgression and gene pyramiding, culminating in PBW723—a line with over 81% recovery of the recurrent parent genome and improved yellow rust resistance, released for commercial cultivation in 2017 (Sharma et al. 2021). Similarly, the transfer of high thousand grain weight (TGW) traits into elite cultivars PBW343 and PBW550, another high-yielding variety developed by PAU and released in 2008 (Gupta et al. 2018), coupled with the pyramiding of yellow rust and leaf rust resistance in PBW550, demonstrates the synergy between modern molecular tools and traditional breeding approaches (Kaur et al. 2020).

While MAS has proven highly effective for traits governed by major genes or with tightly linked markers, it faces limitations when dealing with QTLs due to their small effects and complex genotype-by-environment interactions. To address these challenges, GS has emerged as a more effective tool for addressing complex polygenic traits.

### Genomic selection (GS) and the power of predictive breeding

GS builds upon MAS by incorporating genome-wide markers into predictive breeding models (Meuwissen et al. 2001). Unlike MAS, which relies on a few selected markers, GS evaluates entire genomic profiles to estimate breeding values for polygenic traits such as quantitative disease resistance, drought tolerance, and yield stability. The effectiveness of GS depends on the accuracy of genomic estimated breeding value (GEBV), which varies across models such as ridge regression (RR-BLUP), Bayesian regression (BayesCπ), and deep learning-based neural networks. Incorporating functional markers, epistatic interactions, and environment-specific genomic prediction models has further improved selection accuracy for wheat breeding (Clark and van der Werf 2013).

A key advantage of GS is its ability to shorten breeding cycles by predicting trait performance before field trials are conducted (Heffner et al. 2010; Cerrudo et al. 2018). However, long-term genetic variance reduction observed in some GS-based programmes highlights the risk of narrowing diversity. To mitigate this, strategies such as optimised training populations and multi-year data calibration are essential (Rutkoski et al. 2015). Recent applications of GS in wheat have demonstrated its potential for enhancing disease resistance breeding. For instance, studies at CIMMYT have shown that GS-based selection for APR to stem rust improves prediction accuracy when *Sr2-*linked markers are included as fixed effects in G-BLUP models (Rutkoski et al. 2014). Additionally, GS models trained on multi-environment datasets have shown higher predictive accuracies for yellow rust and powdery mildew resistance, highlighting the importance of integrating environmental data into GS pipelines (Ornella et al. 2012).

Despite its success, GS still faces challenges in wheat due to its hexaploid genome, which introduces complexity in capturing rare alleles from the D sub-genome that has been historically constrained by genetic bottlenecks. Origin-specific genomic selection (OSGS) addresses this limitation by distinguishing between exotic and elite allele sources and applying differential weighting to avoid penalising rare, beneficial alleles (Yang et al. 2020). This approach has been successfully demonstrated in maize and barley, and holds promise for application in wheat, particularly in pre-breeding and introgression efforts aimed at retaining favourable alleles from wild or landrace donors.

### CRISPR/Cas genome editing (GE): a new era for precision breeding

GE has revolutionised breeding by enabling precise modifications—deletions, insertions, and point mutations—in endogenous genes. This technology offers a novel strategy for disease resistance breeding. Recent applications in wheat have primarily focused on modifying disease susceptibility genes (Su et al. 2011; Liu et al. 2024). Simultaneous editing of three homoeoalleles of *TaMLO-A1* or *TaEDR1* have demonstrated enhanced resistance to powdery mildew (Wang et al. 2014; Zhang et al. 2017), while inactivation of *TaGW2-6A*, a key regulator of TGW, has increased yellow rust resistance by altering the plant’s defence response pathways (Liu et al. 2024).

A major breakthrough in GE-based disease resistance is the development of *Tamlo-R32*, a wheat mutant carrying a 304-kb deletion at the *TaMLO-B1* locus, which confers durable resistance to powdery mildew. Unlike previous *mlo-*knockout lines, *Tamlo-R32* maintains normal growth and yield potential due to ectopic activation of *TaTMT3B*, an alternative sugar transporter gene that compensates for the pleiotropic effects of *mlo* loss-of-function mutations (Li et al. 2022). While *Tamlo-R32* represents a significant step towards commercial application, it also underscores the complexity of engineering durable disease resistance in wheat.

Further developments such as base editing, prime editing, and multiplexed CRISPR editing are expanding GE applications by allowing the targeting of multiple resistance genes, overcoming the limitations of traditional introgression-based breeding methodologies (Zhang et al. 2018d; Zhang et al. 2019; Li et al. 2020c; Zhang et al. 2021b; Ni et al. 2023; Ricroch et al. 2024; Yu et al. 2024; Abdallah et al. 2025). Base editing allows targeted alteration of single base pairs without generating double strand breaks, minimising genomic instability and off-target effects. Prime editing refines this precision by enabling all 12 possible base conversions, in addition to controlled insertions and deletions.

Beyond technical advancements, the regulatory context is also shifting in ways that favour GE-based breeding. Unlike traditional transgenic approaches, which involve the stable insertion of foreign DNA and remain tightly regulated as genetically modified organisms (GMOs), GE allows for precise, transgene-free modifications and is increasingly treated under distinct regulatory frameworks. Edits made using site-directed nucleases (SDNs) are typically classified into three types: SDN-1 introduces small insertions or deletions without a DNA template; SDN-2 uses a short template to make precise changes like point mutations; and SDN-3 inserts larger, often foreign DNA. Provided no foreign DNA remains in the final product, SDN-1 and SDN-2 edits are now exempt from GMO regulation in the USA, India, and England (Hundleby and Harwood 2022; UK Parliament 2023). The “New Genomic Techniques” (NGT) regulatory framework, under negotiation in the European Union, is expected to exempt lightly edited Category 1 NGT plants, including those developed using SDN-1 and some SDN-2 approaches, from GMO regulations, pending final approval (Mundorf et al. 2025). In the majority of cases SDN-3 plants remain strictly regulated. These global regulatory shifts are critical as they increasingly facilitate the deployment of GE for disease resistance, yield improvement, and quality traits.

Although GE-based disease resistance traits in wheat are still in the early stages of commercialisation, the technology has already been adopted for other crop traits, highlighting its broader potential. Rice varieties DRR-Dhan 100 (Kamala) and Pusa DST Rice 1, developed using CRISPR/Cas9, offer enhanced drought and salt tolerance, increased yield, and have recently been released for cultivation in India (Priyadarshini 2025). Other gene-edited crops, including high-oleic soybeans (ISAAA 2019a) developed by Pennsylvania State University and Calyxt, along with Intrexon’s non-browning GreenVenus™ Romaine lettuce (ISAAA 2019b), have successfully reached markets in the USA and Japan. These examples illustrate GE’s versatility and growing acceptance, suggesting that traits for disease resistance are likely to become similarly successful.

One of the most promising applications of GE in wheat breeding is its ability to overcome linkage drag. Resistance genes tightly linked to undesirable agronomic traits, due to recombination suppression in introgressed segments, pose a significant challenge when transferring genes from homoeologous chromosomes into wheat. GE offers a solution by enabling the inactivation or knockout of susceptibility loci within elite genetic backgrounds (Zafar et al. 2020; Jin et al. 2022) or by accelerating the domestication of wild relatives through targeted editing of key traits that enhance their agronomic suitability (Lemmon et al. 2018; Zsögön et al. 2018). Using conventional breeding, this would require multiple rounds of repeated backcrossing. Additionally, GE facilitates the pyramiding of R genes with diverse mechanisms of action into a single variety, enhancing durability and effectiveness and providing robust, long-lasting resistance against field pathogen populations.

Despite these technological advancements, all targeted breeding approaches require prior knowledge of target genes or QTLs and closely linked effective markers. Markers, particularly in the case of the D-genome, developed from elite wheat materials, may not fully reflect genetic variation in non-adapted germplasm, thus bespoke marker platforms for wild relatives are required. The integration of MAS, GS, deep learning, and GE has reshaped what is possible in wheat breeding, improving the applicability and usefulness of ancestral resistance genes. However, while these technologies accelerate breeding progress, they also present limitations that must be addressed to fully realise their potential in securing global food production.

## Conclusions

Centuries of refinement through domestication and selective breeding have narrowed the genetic base of modern wheat, inadvertently reducing its resilience to evolving biotic stressors. In contrast, wild relatives and ancestral wheat germplasm retain extensive allelic diversity, representing an underexploited reservoir of disease resistance genes with significant potential for crop improvement.

While the advent of MAS, GS, and GE has transformed resistance breeding by enabling the precise stacking, transfer, and modification of target loci, challenges remain regarding scalability, regulatory acceptance, and integration into conventional breeding pipelines. Future strategies must emphasise the development of broad-spectrum resistance, capable of targeting multiple pathogens, while preserving allelic diversity within introgressed genomic regions. Emerging technologies, including deep learning, predictive genomics, and real-time pathogen surveillance, offer novel avenues for anticipatory resistance breeding. In parallel, approaches such as synthetic gene pyramiding and the directed evolution of resistance genes hold promise for enhancing both durability and functional versatility.

The strategic integration of these advanced tools with continued utilisation of genetic diversity from the primary, secondary, and tertiary gene pools will be critical. Achieving resilient, high-yielding wheat cultivars capable of withstanding the combined challenges of emerging pathogens and climate-induced stress will require a holistic, forward-looking approach to breeding.

Historically, fungicides have played a critical role in protecting wheat yields and mitigating disease pressure. However, the widespread emergence of fungicide-resistant pathogen populations now threatens the long-term efficacy of chemical control measures. This reinforces the urgent need to identify, characterise, and deploy novel genetic resistance sources as a foundational component of integrated disease management strategies. In this context, renewed exploration of underutilised germplasm and the application of precision breeding technologies will be increasingly critical. As disease pressures evolve and pathogen phenology shifts, while chemical controls continue to lose efficacy, the long-term sustainability of wheat production will increasingly rely on durable resistance, underpinned by enhanced genetic diversity and enabled by technological innovation.
